# Extracellular Vesicle-Derived MicroRNAs as Early Diagnostic Biomarkers of Diabetic Nephropathy and Cardiovascular Diseases in Type 2 Diabetes

**DOI:** 10.3390/ijms27125581

**Published:** 2026-06-20

**Authors:** Yessenbekova Arailym, Arman Abaildayev, Belkozhayev Ayaz

**Affiliations:** 1 Department of Biophysics, Biomedicine and Neuroscience, Al-Farabi Kazakh National University, Al-Farabi Av. 71, Almaty 050040, Kazakhstan; arailym.yesenbekova@kaznu.kz; 2 Department of Chemical and Biochemical Engineering, Geology and Oil-Gas Business Institute Named After K. Turyssov, Satbayev University, Almaty 050043, Kazakhstan; 3 Department of Molecular Biology and Genetics, Al-Farabi Kazakh National University, Al-Farabi Av. 71, Almaty 050040, Kazakhstan

**Keywords:** extracellular vesicles, exosomes, microRNA, type 2 diabetes mellitus, biomarkers, liquid biopsy, miR-21, miR-192, atherosclerosis, clinical translation

## Abstract

Type 2 diabetes mellitus (T2DM) is a major driver of chronic kidney disease and cardiovascular morbidity worldwide. Extracellular vesicles (EVs), particularly exosomes, carry microRNAs (miRNAs) that reflect the pathophysiological state of their parent cells and represent promising non-invasive biomarkers. This review comprehensively examines the diagnostic and mechanistic roles of EV-derived miRNAs in diabetic nephropathy (DN) and cardiovascular diseases (CVDs) associated with T2DM. A PRISMA-guided literature search of PubMed, Scopus, Web of Science, and Embase identified 847 articles published between January 2020 and June 2026, of which 156 studies met the inclusion criteria. Several urinary exosomal miRNAs demonstrated significant diagnostic performance for DN, including miR-4534 (AUC = 0.786), miR-136-5p (sensitivity 72.2%, specificity 78.4%), and miR-142-3p. A meta-analysis of circulating miRNAs in diabetic kidney disease reported a pooled AUC of 0.79. In the cardiovascular setting, exosomal miR-155-5p (AUC = 0.901), miR-15a-3p (AUC = 0.874), and a four-miRNA panel (miR-433-3p/let-7b/miR-30-5p/miR-122-5p; AUC = 0.833) demonstrated strong diagnostic performance for ischemic heart disease and carotid atherosclerosis in T2DM. Mechanistically, key EV-associated miRNAs, including miR-21, miR-192, and the anti-fibrotic miR-29 family, participate in fibrosis, inflammation, oxidative stress, endothelial dysfunction, and cardiac remodeling pathways. EV-derived miRNAs therefore represent highly promising non-invasive biomarkers for the early diagnosis and monitoring of diabetic renal and cardiovascular complications. However, clinical translation requires standardized EV isolation and miRNA detection protocols, together with validation in large multicenter prospective cohorts. This review highlights the considerable diagnostic and translational potential of EV-derived miRNAs for precision medicine and liquid biopsy applications in T2DM complications.

## 1. Introduction

T2DM has emerged as one of the most pressing global health challenges of the twenty-first century, affecting over 530 million adults worldwide, with projections indicating a rise to 783 million by 2045. The chronic hyperglycemic milieu in T2DM initiates a cascade of metabolic derangements that culminate in devastating microvascular and macrovascular complications, among which DN and CVDs constitute the leading causes of morbidity and mortality [1,2]. DN, also termed diabetic kidney disease (DKD), develops in approximately 30–40% of patients with T2DM and represents the single most common etiology of end-stage renal disease globally. Cardiovascular complications, including coronary artery disease, heart failure, and stroke, occur two to four times more frequently in individuals with T2DM compared to their non-diabetic counterparts, accounting for approximately 50–80% of deaths in this population [3,4].

Current diagnostic paradigms for DN rely predominantly on the measurement of albuminuria and estimated glomerular filtration rate (eGFR), both of which have well-recognized limitations. Albuminuria, while serving as the earliest conventional marker, may be absent in up to 30% of patients who exhibit histological evidence of diabetic kidney injury, a phenotype termed non-albuminuric DKD. Furthermore, albuminuria is influenced by numerous confounding factors, including exercise, dietary protein intake, fever, and infection, potentially leading to false-positive results [1,5]. Similarly, eGFR decline often becomes clinically apparent only after substantial irreversible nephron loss has occurred, underscoring the inadequacy of these biomarkers for early disease detection. In the cardiovascular domain, traditional risk stratification tools such as the Framingham Risk Score inadequately capture the excess cardiovascular risk attributable to T2DM, frequently underestimating event probability in diabetic cohorts [1,2].

The imperative for novel, non-invasive biomarkers capable of detecting diabetic complications at their earliest, potentially reversible stages has catalyzed intense research into the liquid biopsy paradigm. Among the most promising candidates to emerge from this effort are extracellular vesicle-derived microRNAs (EV-miRNAs). EVs are membrane-bound nanoparticles released by virtually all cell types into the extracellular space and biological fluids, including blood, urine, saliva, and cerebrospinal fluid [6,7]. These vesicles serve as sophisticated vehicles for intercellular communication, transporting a diverse cargo of proteins, lipids, and nucleic acids—most notably microRNAs (miRNAs)—from donor to recipient cells, thereby modulating gene expression and cellular phenotype at both local and distant sites [8,9,10,11,12,13,14].

miRNAs are small, non-coding RNA molecules of approximately 19–25 nucleotides in length that regulate gene expression post-transcriptionally through binding to the 3′ untranslated region (3′-UTR) of target messenger RNAs (mRNAs), leading to mRNA degradation or translational repression [15,16]. Over 2600 mature human miRNAs have been catalogued, and it is estimated that miRNAs collectively regulate more than 60% of protein-coding genes. Critically, miRNAs encapsulated within EVs are shielded from degradation by RNases in biological fluids owing to the protective lipid bilayer membrane of the vesicle, rendering them remarkably stable biomarkers amenable to clinical measurement [17,18]. This inherent stability, combined with the tissue-specific expression patterns of miRNAs and the ability of EVs to reflect the pathophysiological state of their cells of origin, positions EV-miRNAs as ideal candidates for early diagnostic biomarkers [19,20].

In the context of DN, urinary EVs are of particular interest because they originate predominantly from cells lining the nephron-podocytes, tubular epithelial cells, and collecting duct cells—thereby providing a direct “window” into the renal microenvironment without the need for invasive tissue biopsy [5,21]. Several EV-derived miRNAs, including miR-21, miR-192, miR-29 family members, miR-4534, miR-136-5p, and miR-663a, have been implicated in key pathogenic mechanisms of DN, such as TGF-β-mediated fibrosis, epithelial-to-mesenchymal transition (EMT), podocyte apoptosis, and inflammasome activation [22,23,24,25]. A recent meta-analysis encompassing 24 studies and over 3000 participants reported a pooled area under the receiver operating characteristic curve (AUC) of 0.79, with sensitivity of 0.76 and specificity of 0.74, for circulating miRNAs in the diagnosis of DKD [26,27,28].

In the cardiovascular arena, EV-miRNAs have demonstrated compelling diagnostic and prognostic utility across the spectrum of diabetic cardiovascular complications, from subclinical atherosclerosis to acute myocardial infarction and heart failure. Exosomal miR-155-5p has achieved an AUC of 0.901 for the diagnosis of ischemic heart disease in diabetic patients [29], while a four-miRNA serum exosomal panel (miR-433-3p, let-7b, miR-30-5p, and miR-122-5p) demonstrated a combined AUC of 0.833 for carotid atherosclerosis detection in T2DM [30]. Moreover, mechanistic studies have elucidated how EV-miRNAs mediate intercellular crosstalk in the diabetic heart, exemplified by the discovery that diabetic cardiomyocyte-derived small EVs carry reduced levels of miR-194-3p, leading to enhanced TGFβR2-mediated fibroblast-to-myofibroblast conversion and cardiac fibrosis [31,32,33,34,35].

Despite the considerable promise of EV-miRNAs as biomarkers, significant challenges impede their clinical translation. These include the lack of standardized protocols for EV isolation and characterization, the absence of universally accepted reference genes for miRNA normalization, the heterogeneity of study designs and patient populations, and the need for validation in large-scale prospective multicenter trials [36,37,38]. The International Society for EVs has published updated guidelines (MISEV2023) to address some of these concerns, but substantial work remains before EV-miRNA biomarkers can be integrated into routine clinical practice [37,39,40].

This comprehensive review aims to systematically examine the current evidence on EV-derived miRNAs as early diagnostic biomarkers of DN and CVDs in the setting of T2DM. We provide an updated synthesis of the biogenesis and classification of EVs, the mechanisms of miRNA sorting into EVs, the pathogenic roles of specific EV-miRNAs in DN and CVD, their diagnostic performance as biomarkers, and the methodological considerations surrounding EV isolation and miRNA detection. Furthermore, we critically appraise the challenges facing clinical translation and propose future research directions to accelerate the bench-to-bedside journey of these promising biomarkers.

## 2. Materials and Methods

### 2.1. Search Strategy

**Figure 1:**
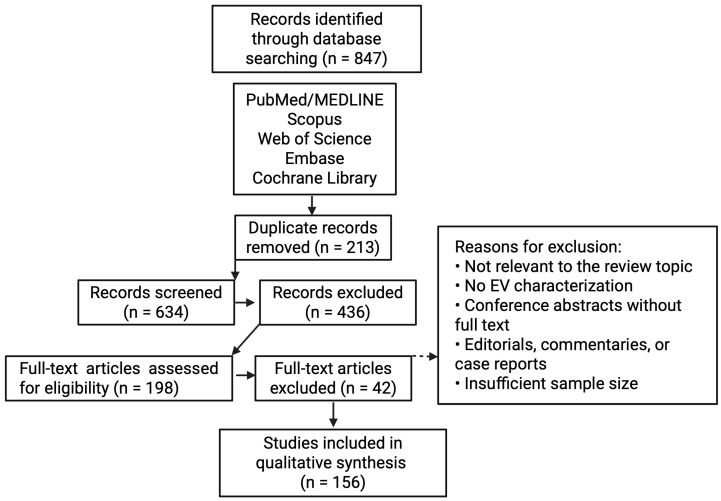
PRISMA 2020 flow diagram of the literature search, screening, eligibility assessment, and study selection process. Note: The dashed arrow indicates the relationship between the excluded full-text articles and the corresponding reasons for exclusion. Created in BioRender.com. Belkozhayev A. (2026), License No. DC29SFCYEA.

A comprehensive literature search was conducted in accordance with the Preferred Reporting Items for Systematic Reviews and Meta-Analyses (PRISMA) guidelines. The following electronic databases were systematically searched: PubMed/MEDLINE, Scopus, Web of Science, Embase, and the Cochrane Library. The search was restricted to articles published between 1 January 2020, and 30 June 2026. The search strategy employed a combination of Medical Subject Headings (MeSH) terms and free-text keywords, including: “ EVs “ OR “exosomes” OR “microvesicles” AND “microRNA” OR “miRNA” OR “miR” AND “diabetic nephropathy” OR “diabetic kidney disease” OR “DKD” OR “cardiovascular disease” OR “diabetic cardiomyopathy” OR “atherosclerosis” OR “coronary artery disease” OR “heart failure” AND “type 2 diabetes” OR “T2DM” OR “diabetes mellitus” AND “biomarker” OR “diagnosis” OR “diagnostic”. Reference lists of included studies and relevant review articles were manually screened to identify additional eligible publications. A PRISMA 2020 flow diagram summarizing the study selection process is provided in Figure 1. This review was conducted according to PRISMA 2020 recommendations; however, the review protocol was not prospectively registered in PROSPERO or any other review registry.

### 2.2. Inclusion and Exclusion Criteria

Inclusion criteria encompassed: (1) original research articles (clinical studies, preclinical studies with translational relevance), systematic reviews, and meta-analyses; (2) studies investigating miRNAs derived from or associated with EVs; (3) studies examining diabetic nephropathy, DKD, or CVDs in the context of T2DM; (4) studies reporting diagnostic biomarker data (AUC, sensitivity, specificity) or pathogenic mechanistic data; (5) articles published in English in peer-reviewed journals. Exclusion criteria included: (1) studies focused exclusively on type 1 diabetes or gestational diabetes; (2) studies examining cell-free miRNAs without EV characterization; (3) conference abstracts without full-text availability; (4) case reports, editorials, and commentaries without original data; (5) studies with sample sizes fewer than 5 per group; (6) duplicate publications.

### 2.3. Data Extraction and Quality Assessment

Two independent reviewers screened titles and abstracts, followed by full-text evaluation of potentially eligible studies. Data were extracted using a standardized form capturing study design, sample type (serum, plasma, urine), patient demographics, EV isolation method, miRNA detection platform, identified miRNAs, regulation direction, diagnostic performance metrics, target genes and pathways, and study quality indicators. Discrepancies were resolved by consensus with a third reviewer. The Quality Assessment of Diagnostic Accuracy Studies 2 (QUADAS-2) tool was employed for assessing the risk of bias in diagnostic accuracy studies. A total of 847 records were initially identified through database searching. After removal of duplicate records (*n* = 213), 634 articles remained for title and abstract screening. Following screening, 198 full-text articles were assessed for eligibility, 156 studies met the inclusion criteria and were included in this review. Of these, 42 articles were excluded because they did not meet the predefined inclusion criteria. Ultimately, 156 studies were included in the qualitative synthesis. The study selection process is summarized in the PRISMA 2020 flow diagram (Figure 1).

## 3. Biogenesis and Classification of EVs

EVs represent a heterogeneous family of membrane-enclosed particles released by virtually all cell types under both physiological and pathological conditions. The recognition that EVs serve as fundamental mediators of intercellular communication, rather than mere cellular debris, has revolutionized our understanding of cell biology and opened new frontiers in biomarker discovery and therapeutic development [6,7,41]. The International Society for Extracellular Vesicles (ISEV) has established nomenclature guidelines through the MISEV2018 and updated MISEV2023 position statements, which recommend classification based on physical characteristics, biochemical composition, and cellular origin rather than biogenesis pathway alone, given the current technical limitations in definitively assigning individual vesicles to specific biogenesis routes [37,42,43].

### 3.1. Exosomes (30–150 nm)

Exosomes represent the best-characterized subclass of EVs and have garnered the most attention in the biomarker field. These nano-sized vesicles (30–150 nm in diameter) originate through the endosomal pathway. The biogenesis of exosomes begins with the inward budding of early endosomal membranes to form intraluminal vesicles (ILVs) within late endosomes, thereby generating multivesicular bodies (MVBs). Upon fusion of MVBs with the plasma membrane, ILVs are released into the extracellular space as exosomes [6,44,45,46].

Two principal molecular machineries govern ILV formation within MVBs: the ESCRT-dependent pathway and ESCRT-independent pathways. The Endosomal Sorting Complex Required for Transport (ESCRT) consists of four multiprotein complexes (ESCRT-0, -I, -II, -III) plus associated proteins such as VPS4, TSG101, ALIX (also termed PDCD6IP), and HRS. ESCRT-0 recognizes and clusters ubiquitinated cargo on the endosomal membrane; ESCRT-I and ESCRT-II promote membrane deformation and invagination; ESCRT-III executes membrane scission and ILV release into the MVB lumen; and VPS4 ATPase disassembles the ESCRT-III complex for recycling [47]. The ESCRT machinery is also responsible for the selective sorting of specific protein and nucleic acid cargo into ILVs.

ESCRT-independent pathways of exosome biogenesis involve ceramide generation by neutral sphingomyelinase 2 (nSMase2), which converts sphingomyelin to ceramide in the endosomal membrane. Ceramide, a cone-shaped lipid, spontaneously promotes negative membrane curvature and ILV budding without the need for ESCRT proteins [48,49]. The tetraspanin family of transmembrane proteins, particularly CD63, CD9, and CD81, also facilitates ESCRT-independent exosome biogenesis by organizing membrane microdomains enriched in specific lipids and proteins that promote ILV formation. These tetraspanins are abundantly expressed on exosome surfaces and serve as widely used molecular markers for exosome identification and isolation [50]. Additional ESCRT-independent mechanisms involving syndecan-syntenin-ALIX, phospholipase D2, and RAB31-flotillin have been described, highlighting the remarkable diversity and redundancy of exosome biogenesis pathways.

The fate of MVBs is determined by a balance between fusion with the plasma membrane (leading to exosome release) and fusion with lysosomes (leading to cargo degradation). RAB GTPases, particularly RAB27A, RAB27B, RAB11, and RAB35, regulate MVB trafficking to the cell surface and exosome secretion. SNARE proteins (including VAMP7, YKT6, and syntaxin-5) mediate the final fusion event between MVBs and the plasma membrane [45,50]. The regulation of exosome secretion is influenced by numerous cellular stimuli, including calcium influx, oxidative stress, hypoxia, p53 activation, and, notably, hyperglycemia—a finding of particular relevance to the diabetic milieu.

### 3.2. Microvesicles (100–1000 nm)

Microvesicles (MVs), also referred to as ectosomes, microparticles, or shedding vesicles, are generated by direct outward budding and fission of the plasma membrane. This process is mechanistically distinct from exosome biogenesis and involves cytoskeletal reorganization, increases in intracellular calcium concentration, and the activity of enzymes such as flippases, floppases, and scramblases that disrupt the normal asymmetric distribution of phospholipids across the plasma membrane bilayer [6,51]. The externalization of phosphatidylserine to the outer leaflet of the membrane is a hallmark of MV formation and serves as a molecular signal for recognition and uptake by recipient cells.

Signaling molecules including ARF6, RHOA, and RAC1 GTPases regulate actin dynamics during MV budding. The ARRDC1-TSG101 interaction links ESCRT-I to the plasma membrane, facilitating MV release in certain contexts. Microvesicles range in size from approximately 100 nm to 1000 nm, although considerable size overlap with exosomes in the 100–200 nm range poses technical challenges for their discrimination. MVs carry surface markers reflective of their parent cell, including integrins, selectins, tissue factor, and CD40 ligand, along with cytoplasmic cargo comprising proteins, mRNAs, and miRNAs [6,34]. In the context of diabetes, platelet-derived microvesicles have been shown to be elevated and contribute to the prothrombotic state, while endothelial-derived MVs serve as markers of vascular injury and dysfunction [6,52,53,54].

### 3.3. Apoptotic Bodies (1000–5000 nm)

Apoptotic bodies (ApoBDs) are the largest subclass of EVs, generated during the late stages of programmed cell death through membrane blebbing and cellular fragmentation. These vesicles typically range from 1000 to 5000 nm in diameter and contain nuclear fragments, organelles, DNA, histones, and cytoplasmic constituents. Phosphatidylserine exposure on their surface facilitates recognition and phagocytic clearance by macrophages and neighboring cells, which is critical for preventing the release of intracellular contents that could trigger inflammatory responses [6,44]. While traditionally viewed primarily as disposal vehicles, emerging evidence suggests that ApoBDs can transfer functional molecules, including miRNAs, to recipient cells, thereby influencing cellular behavior and contributing to tissue homeostasis or disease progression.

### 3.4. EV Secretion in the Diabetic Milieu

The hyperglycemic environment characteristic of T2DM profoundly influences EV biogenesis, secretion, and cargo composition. Multiple studies have demonstrated that chronic hyperglycemia, advanced glycation end-products (AGEs), oxidative stress, and inflammatory mediators significantly increase EV release from various cell types relevant to diabetic complications, including renal cells, endothelial cells, cardiomyocytes, and immune cells [55,56]. High-glucose conditions have been shown to enhance exosome secretion from podocytes, mesangial cells, and tubular epithelial cells, with the released EVs carrying altered miRNA and protein cargo that propagates pathogenic signaling to neighboring and distant cells [21,22].

Mechanistically, hyperglycemia-induced oxidative stress activates the NLRP3 inflammasome, which not only promotes the release of pro-inflammatory cytokines (IL-1β and IL-18) but also enhances EV secretion. The AGE-RAGE (receptor for advanced glycation end-products) axis further stimulates EV production through downstream activation of NF-κB and MAPK signaling pathways. Additionally, the diabetic milieu alters the lipid composition of EV membranes, increasing ceramide content and modifying cholesterol ratios, which in turn affects EV stability, fusogenicity, and uptake by recipient cells [55,57,58]. These diabetes-induced alterations in EV biology create a pathogenic EV landscape that actively contributes to the progression of DN and cardiovascular disease, while simultaneously offering opportunities for biomarker development. A comparative overview of EV subtypes, their biogenesis pathways, surface markers, and molecular cargo is presented in (Table 1).

### 3.5. miRNA Biogenesis and Loading into EVs

#### 3.5.1. Canonical miRNA Biogenesis Pathway

The biogenesis of miRNAs follows a precisely orchestrated multi-step pathway that begins in the nucleus and concludes in the cytoplasm. In the canonical pathway, miRNA genes are transcribed predominantly by RNA polymerase II to generate primary miRNA transcripts (pri-miRNAs), which are typically several kilobases in length and contain one or more hairpin structures. The nuclear microprocessor complex, comprising the RNase III enzyme Drosha and its cofactor DGCR8 (DiGeorge Syndrome Critical Region Gene 8), recognizes and cleaves the pri-miRNA hairpin to release a ~70-nucleotide precursor miRNA (pre-miRNA) with a characteristic stem-loop structure and 2-nucleotide 3′ overhang [15,16,59].

The pre-miRNA is subsequently exported from the nucleus to the cytoplasm by Exportin-5 in a Ran-GTP-dependent manner. In the cytoplasm, the RNase III enzyme Dicer, in complex with the transactivation-responsive RNA-binding protein (TRBP), cleaves the terminal loop of the pre-miRNA to generate a ~22-nucleotide double-stranded miRNA duplex. One strand (the guide strand or mature miRNA) is preferentially loaded into an Argonaute (AGO) protein (predominantly AGO2 in mammals) within the RNA-induced silencing complex (RISC), while the passenger strand (miRNA*) is typically degraded [15,60]. The mature miRNA-RISC complex then guides sequence-specific recognition and binding to complementary sites in the 3′-UTR of target mRNAs, leading to translational repression, mRNA deadenylation, or direct mRNA cleavage.

Non-canonical miRNA biogenesis pathways, including Drosha-independent (mirtrons processed by the spliceosome) and Dicer-independent (miR-451 processed by AGO2 catalytic activity) mechanisms, contribute a minority of mature miRNAs but may be relevant in specific pathological contexts. The expression of individual miRNAs is regulated at multiple levels: transcriptional control by transcription factors and epigenetic modifications, processing efficiency by RNA-binding proteins and RNA modifications (m6A methylation), and turnover rates determined by target-mediated degradation and exonuclease activity [16]. These regulatory layers ensure precise spatiotemporal control of miRNA expression, and their dysregulation in diabetes contributes to the altered miRNA profiles observed in diabetic tissues and circulating EVs.

#### 3.5.2. Selective Sorting of miRNAs into EVs

The miRNA content of EVs does not simply mirror the intracellular miRNA landscape; rather, specific miRNAs are selectively enriched or depleted in EVs relative to their parent cells, indicating the existence of active sorting mechanisms. Several key molecular determinants of miRNA sorting into EVs have been identified. The heterogeneous nuclear ribonucleoprotein A2B1 (hnRNPA2B1) recognizes specific sequence motifs (EXO-motifs, such as GGAG and CCCU) in miRNAs and directs their loading into exosomes. Notably, hnRNPA2B1 in exosomes is sumoylated, and this post-translational modification is essential for its miRNA-sorting activity [61,62,63].

SYNCRIP (synaptotagmin-binding cytoplasmic RNA-interacting protein) binds to the GGCU motif in miRNAs and facilitates their loading into hepatocyte-derived exosomes, demonstrating that sorting mechanisms can be cell-type-specific [58]. The Y-box protein 1 (YBX1) has been implicated in the sorting of miR-223 into exosomes, while the RNA-binding protein major vault protein (MVP) facilitates selective sorting of miR-193a. Additionally, the ceramide-dependent pathway of exosome biogenesis influences miRNA loading, as inhibition of neutral sphingomyelinase 2 (nSMase2) reduces the secretion of specific miRNAs in exosomes [48,64].

A landmark study revealed that miRNA sequence features, specifically 3′ end nucleotide composition, encode information that determines whether a miRNA is preferentially retained within cells or exported via small EVs. MiRNAs enriched in cells tended to have specific 3′ sequence codes distinct from those enriched in EVs, suggesting a previously unappreciated layer of miRNA sorting regulation [65]. Furthermore, uridylation and adenylation of the 3′ end of miRNAs serve as post-transcriptional modifications that influence sorting: uridylated miRNAs are enriched in EVs, whereas adenylated isoforms are preferentially retained within cells [66,67].

The selective sorting of miRNAs into EVs has profound implications for biomarker development. It means that EV-miRNA profiles can provide information about cellular processes that may not be captured by measuring total circulating miRNAs. In the diabetic context, hyperglycemia has been shown to alter the sorting machinery itself, modifying the repertoire of miRNAs packaged into EVs released by podocytes, tubular cells, cardiomyocytes, and endothelial cells [55,57,68]. Understanding these sorting mechanisms is therefore critical not only for interpreting EV-miRNA biomarker data but also for developing EV-based therapeutic delivery strategies.

#### 3.5.3. Stability of EV-Enclosed miRNAs in Biological Fluids

One of the most compelling attributes of EV-miRNAs as clinical biomarkers is their extraordinary stability in biological fluids. Extracellular miRNAs exist in several forms: encapsulated within EVs, bound to AGO2 protein complexes, or associated with high-density lipoproteins (HDL). EV-enclosed miRNAs are particularly resistant to degradation because the lipid bilayer membrane of the vesicle provides robust protection against endogenous RNases, extreme pH, and repeated freeze–thaw cycles [17,18]. Studies have demonstrated that EV-derived miRNAs remain detectable and quantifiable in urine samples stored at −80 °C for over 5 years, and in serum/plasma samples subjected to multiple freeze–thaw cycles, with minimal degradation compared to cell-free miRNAs.

The stability of urinary exosomal miRNAs is particularly relevant for DN biomarker applications. Urinary EVs, primarily originating from renal epithelial cells lining the nephron, carry a miRNA cargo that directly reflects the molecular state of the kidney. The protective lipid bilayer ensures that these kidney-derived miRNAs survive the transit through the urinary tract and remain intact for downstream analysis [5,21]. This stability, combined with the non-invasive nature of urine collection, makes urinary EV-miRNAs particularly attractive for longitudinal monitoring of disease progression and treatment response in clinical settings [69].

### 3.6. EV-miRNAs in the Pathogenesis of DN

The pathogenesis of DN involves a complex interplay of hemodynamic, metabolic, and inflammatory mechanisms that collectively drive glomerular hyperfiltration, mesangial expansion, basement membrane thickening, podocyte loss, tubulointerstitial fibrosis, and progressive decline in renal function. EV-mediated transfer of miRNAs between renal cell populations has emerged as a critical mechanism through which these pathogenic processes are coordinated and amplified in the diabetic kidney [12,13,14,70,71,72,73].

#### 3.6.1. miR-21: The Master Fibrotic Regulator

miR-21 is arguably the most extensively studied miRNA in the context of DN and occupies a central position in the fibrotic signaling network. In the diabetic kidney, miR-21 expression is markedly upregulated in podocytes, mesangial cells, and tubular epithelial cells under the influence of TGF-β1, the master profibrotic cytokine in DN. EV-mediated transfer of miR-21 between renal cell types amplifies fibrotic signaling in a paracrine and autocrine manner [74,75,76].

The pathogenic actions of miR-21 in DN are mediated primarily through suppression of two key tumor suppressors: PTEN (phosphatase and tensin homolog) and SMAD7. By targeting PTEN, miR-21 activates the PI3K/Akt signaling cascade, which promotes mesangial cell proliferation, hypertrophy, and extracellular matrix (ECM) production. The PTEN/PI3K/Akt axis also inhibits podocyte autophagy, a cytoprotective mechanism, thereby rendering podocytes vulnerable to apoptosis under hyperglycemic stress [75,77]. Simultaneously, miR-21-mediated suppression of SMAD7, an inhibitory SMAD that normally counterbalances TGF-β/SMAD3 signaling, creates a positive feedback loop that perpetuates fibrogenesis. miR-21 has been shown to promote renal fibrosis in DN by targeting both PTEN and SMAD7, leading to enhanced collagen I, collagen IV, and fibronectin deposition in the mesangial matrix [75].

Exosomal transfer of miR-21 from injured tubular epithelial cells to interstitial fibroblasts has been identified as a mechanism of tubulointerstitial fibrosis propagation. In the cardiovascular context, exosomal miR-21 derived from cardiac progenitor cells has demonstrated protective effects against cardiomyocyte apoptosis by targeting programmed cell death 4 (PDCD4), highlighting the context-dependent nature of miR-21 function [78]. Serum exosomal miR-21 levels have been shown to be significantly elevated in patients with acute myocardial infarction and correlate with cardiac troponin levels, suggesting its dual utility as a biomarker in both renal and cardiovascular diabetic complications [11,33,79].

Importantly, the biological functions of miR-21 appear to be highly context-dependent. While numerous studies have linked miR-21 to fibrosis, inflammation, and disease progression in diabetic complications, other reports have demonstrated protective effects, particularly in cardiac repair and cell survival pathways. These apparently conflicting findings likely reflect differences in disease stage, cell type, tissue microenvironment, and experimental models. Therefore, the role of miR-21 should be interpreted within its specific biological context rather than as a uniformly pathogenic or protective regulator.

#### 3.6.2. miR-192: TGF-β/Smad3/EMT Axis

miR-192 is a kidney-enriched miRNA that has been identified as a direct transcriptional target of TGF-β/Smad3 signaling in mesangial cells. In the diabetic kidney, TGF-β1 upregulates miR-192 expression via Smad3 binding to the miR-192 promoter, and the resulting elevation in miR-192 suppresses the transcriptional repressors ZEB1 (zinc finger E-box binding homeobox 1) and ZEB2, thereby de-repressing collagen type I alpha 2 (Col1a2) gene expression and promoting ECM accumulation [23,80]. This TGF-β/Smad3→miR-192→ZEB1/ZEB2→Col1a2 axis represents a critical pathway through which epithelial-to-mesenchymal transition (EMT) and fibrosis are driven in DN.

Urinary exosomal miR-192 levels have been found to be elevated in patients with type 2 diabetes and early microalbuminuria compared to diabetic patients without nephropathy and healthy controls, suggesting its potential as an early biomarker of DN onset. In vitro studies have demonstrated increased miR-192 expression in exosomes secreted by human renal tubular epithelial cells under high glucose conditions, further supporting the concept that urinary exosomal miR-192 reflects active renal injury. A comprehensive review consolidated the multifaceted roles of miR-192 in DN, encompassing its regulation of ECM dynamics, podocyte function, tubular cell apoptosis, and inflammatory responses, consolidating its position as a central mediator and potential therapeutic target [22].

However, the role of miR-192 in DN remains complex and may be context-dependent. While numerous studies have reported profibrotic effects mediated through the TGF-β/Smad3 signaling pathway, other investigations have suggested protective functions depending on disease stage, cell type, and experimental conditions. These findings indicate that miR-192 may exert distinct biological effects during different phases of disease progression, highlighting the need for further studies to clarify its context-specific regulatory functions.

#### 3.6.3. miR-29 Family: Anti-Fibrotic Defense

The miR-29 family, comprising miR-29a, miR-29b, and miR-29c, functions as a potent endogenous anti-fibrotic defense mechanism in the kidney. These miRNAs directly target multiple collagen genes, including COL1A1, COL3A1, and COL4A1, as well as other ECM components such as fibrillin-1 and elastin, thereby suppressing excessive ECM production. In DN, miR-29 family members are significantly downregulated, primarily through TGF-β/Smad3-mediated transcriptional repression, resulting in the de-repression of collagen synthesis and progressive renal fibrosis [81,82,83].

miR-29a has been shown to promote nephrin acetylation and ameliorate hyperglycemia-induced podocyte dysfunction, revealing a novel podocyte-protective function beyond its classical anti-fibrotic role [81]. The protective effects of miR-29a have also been linked to modulation of the DKK1/Wnt/β-catenin signaling pathway in diabetic glomeruli [84]. The depletion of miR-29 family members in urinary EVs has been observed in diabetic patients with progressive kidney disease, and restoration of miR-29 levels through exogenous delivery or de-repression strategies has been proposed as a potential therapeutic approach for DN. The relative decrease in EV-associated miR-29 levels may serve as a biomarker reflecting the transition from adaptive to maladaptive fibrotic responses in the diabetic kidney [80].

#### 3.6.4. Additional Pathogenic EV-miRNAs in DN

Studies have shown that various EV-miRNAs play a role in the pathogenesis of DN. For instance: miR-4449, enriched in serum exosomes from DKD patients, has been shown to promote pyroptosis and oxidative stress in renal tubular cells through the PHRF1-mediated pathway and NLRP3 inflammasome activation, representing a novel pro-inflammatory mechanism of EV-mediated kidney injury [76,85,86,87,88].

Urinary exosomal miR-483-5p, which is sorted into EVs via HNRNPA1-mediated mechanisms, targets MAPK1 and TIMP2 to regulate fibrotic processes. Its expression was found to be significantly downregulated in urinary exosomes from biopsy-confirmed DKD patients, with in vitro experiments confirming that reduced miR-483-5p contributes to podocyte apoptosis and epithelial–mesenchymal transition of tubular epithelial cells [89]. miR-145-5p, enriched in urinary exosomes from DKD patients, induces podocyte apoptosis by inhibiting Srgap2 and activating the RhoA/ROCK signaling pathway [90]. These findings collectively underscore the complexity of the EV-miRNA regulatory network in DN, with multiple miRNAs acting in concert to orchestrate fibrosis, inflammation, EMT, and cell death in the diabetic kidney [83,91,92].

Recent research has expanded the understanding of EV-miRNA-mediated intercellular communication in the diabetic kidney. Proximal tubule-derived exosomes have been shown to transfer miR-92a-1-5p to mesangial cells, contributing to mesangial cell injury and matrix expansion [93]. Furthermore, exosomes from tubular epithelial cells exposed to albumin carry miRNAs that promote macrophage polarization toward the M1 pro-inflammatory phenotype, creating a feed-forward loop of inflammation and tissue injury [94]. The emerging picture is one of a complex, multi-directional EV-miRNA communication network within the diabetic kidney, where different renal cell types exchange pathogenic miRNA signals that collectively drive disease progression [76,95].

### 3.7. EV-miRNAs as Diagnostic Biomarkers of DN

The translation of EV-miRNA biology into clinically applicable biomarkers for DN has been the focus of an expanding body of research, with multiple studies reporting promising diagnostic accuracy metrics. The ideal biomarker for DN should be detectable non-invasively, reflect disease activity at its earliest stages (prior to albuminuria onset), correlate with disease severity and progression, and demonstrate adequate sensitivity and specificity for clinical decision-making [24,26,28,40,69,96,97,98,99].

#### 3.7.1. Urinary Exosomal miRNA Biomarkers

Urinary exosomal miRNAs have attracted particular attention because they originate directly from the renal parenchyma, providing a non-invasive reflection of kidney-specific molecular alterations. Microarray-based screening of urinary exosomal miRNAs from patients with type 2 DKD compared to diabetic controls identified miR-4534 as significantly upregulated in DKD patients [5]. ROC analysis demonstrated an AUC of 0.786 (95% CI: 0.607–0.965), and miR-4534 expression positively correlated with microalbuminuria levels (r = 0.5604, *p* = 0.006). Functional analysis revealed that miR-4534 potentially regulates the FoxO signaling pathway through targeting BNIP3, suggesting its involvement in early DKD pathogenesis [5,27,92].

The first evidence that urinary exosomal miR-136-5p is significantly upregulated in DKD and possesses meaningful diagnostic value was reported [26]. In a study of 42 DN patients and 38 T2DM controls, qRT-PCR validation confirmed miR-136-5p upregulation, with ROC analysis yielding an AUC of 0.722, sensitivity of 72.2%, and specificity of 78.4% [100]. Similarly, urinary exosomal miR-663a was identified as a proximal tubular injury biomarker in DKD, with an AUC of 0.71 for distinguishing DKD from diabetic patients without nephropathy, and its expression correlated with eGFR decline [101].

Small RNA next-generation sequencing was employed to comprehensively profile urinary EV small RNAs from patients with T2D with nephropathy (T2D + DN), T2D without nephropathy (T2D-DN), and healthy controls [102]. This analysis revealed a distinct subset of 13 miRNAs and 10 Piwi-interacting RNAs (piRNAs) significantly dysregulated in DN urinary EVs. Notably, miR-151a-3p and miR-182-5p exhibited a unique expression pattern, being downregulated in T2D-DN but upregulated in T2D + DN, demonstrating their effectiveness in distinguishing patients between these two groups (*p* = 0.0051 for miR-151a-3p and *p* = 0.02 for miR-182-5p) [102]. This differential expression pattern suggests these miRNAs may serve as specific biomarkers for the transition from diabetes without kidney involvement to early nephropathy [98].

Using miRNA sequencing on urinary exosomes from biopsy-confirmed DKD patients with gold-standard histological correlation, miR-483-5p was identified as significantly downregulated in DKD urinary exosomes, and in vitro experiments confirmed that DKD-derived urinary exosomes promoted podocyte apoptosis and tubular cell fibrosis, while bioinformatics analysis revealed involvement of apoptosis and fibrosis-related pathways [89]. The use of biopsy-confirmed cohorts is a notable strength, as it eliminates the diagnostic uncertainty inherent in albuminuria-based classification [95].

#### 3.7.2. Serum and Plasma Exosomal miRNA Biomarkers for DN

While urinary EV-miRNAs provide a direct window into the kidney, serum and plasma exosomal miRNAs offer the advantage of reflecting systemic disease activity and cross-organ communication. Serum exosomes from DKD patients were found to carry elevated levels of miR-4449, which promotes pyroptosis and oxidative stress in renal tubular cells through the PHRF1 pathway, with NLRP3 inflammasome activation as a downstream effector [85]. This finding exemplifies the concept that circulating exosomal miRNAs can be both biomarkers and functional mediators of disease [69,99].

#### 3.7.3. Meta-Analytic Evidence

A comprehensive systematic review and meta-analysis of miRNAs as biomarkers in DKD, encompassing 24 studies with a total of 3052 participants, was conducted [26]. The pooled analysis demonstrated an overall AUC of 0.79 (95% CI: 0.75–0.83), sensitivity of 0.76 (95% CI: 0.71–0.80), and specificity of 0.74 (95% CI: 0.68–0.79) for circulating miRNAs in DKD diagnosis [26]. These meta-analytic results provide level 1 evidence supporting the diagnostic utility of miRNAs in DKD, although the heterogeneity in study designs, miRNA species, and detection methods warrants cautious interpretation. The meta-analysis also identified publication bias and suggested that multi-miRNA panels may outperform individual miRNAs in terms of diagnostic accuracy.

Subgroup analyses revealed that urinary miRNAs demonstrated slightly higher specificity than serum/plasma miRNAs for DN diagnosis, likely reflecting the kidney-specific origin of urinary EV cargo. Additionally, studies employing next-generation sequencing for miRNA discovery followed by qRT-PCR validation in independent cohorts showed more robust diagnostic performance than studies relying on candidate miRNA approaches alone. These findings underscore the importance of unbiased discovery strategies and rigorous multi-cohort validation in EV-miRNA biomarker development. The diagnostic performance of these and other key EV-derived miRNAs as biomarkers for DKD is summarized in (Table 2) [40].

### 3.8. EV-miRNAs in the Pathogenesis of CVDs in Type 2 Diabetes

CVDs represent the predominant cause of mortality in patients with T2DM, encompassing a spectrum that includes diabetic cardiomyopathy, coronary artery disease, coronary microvascular dysfunction, peripheral arterial disease, and heart failure. The diabetic myocardium and vasculature are subject to the deleterious effects of hyperglycemia, insulin resistance, dyslipidemia, and chronic low-grade inflammation, all of which converge to promote cardiac remodeling, accelerated atherosclerosis, and endothelial dysfunction [3,4,103]. EV-mediated miRNA transfer has been identified as a key mechanism through which these pathogenic processes are coordinated between cardiomyocytes, endothelial cells, fibroblasts, smooth muscle cells, and immune cells in the diabetic cardiovascular system [32,33,105,106].

#### 3.8.1. miR-320: Exosomal Anti-Angiogenic Signaling

One of the earliest demonstrations of pathogenic EV-miRNA transfer in diabetic cardiovascular disease involved miR-320. Cardiomyocytes under hyperglycemic conditions were shown to release exosomes enriched with miR-320 that are taken up by neighboring cardiac endothelial cells. Within endothelial cells, miR-320 suppresses its target genes, including insulin-like growth factor 1 (IGF-1), heat shock protein 20 (HSP20), and the transcription factor Ets2, leading to impaired endothelial cell proliferation, migration, and tube formation—collectively resulting in an anti-angiogenic phenotype [107]. This mechanism contributes to the reduced coronary microvascular density and impaired angiogenesis observed in the diabetic heart, which are hallmarks of diabetic cardiomyopathy [108].

#### 3.8.2. miR-194-3p: A Novel Mediator of Diabetic Cardiac Fibrosis

A previously unrecognized mechanism of diabetic cardiac fibrosis mediated by cardiomyocyte-derived sEVs was elucidated [31]. Using both high-fat diet-induced and genetic (db/db) type 2 diabetic mouse models, the authors demonstrated that normal cardiomyocyte-derived Myo-sEVs attenuated cardiac fibrosis and improved diastolic function, whereas diabetic cardiomyocyte-derived Myo-sEVs exacerbated fibrosis and worsened diastolic function [31].

Unbiased microRNA array analysis revealed that miR-194-3p was significantly reduced in diabetic Myo-sEVs compared to normal counterparts. Mechanistic investigations demonstrated that miR-194-3p is a novel upstream inhibitor of TGFβR2 expression, blocking the conversion of fibroblasts to myofibroblasts—the key cellular event in cardiac fibrosis. In diabetes, the reduced delivery of miR-194-3p from cardiomyocytes to fibroblasts via sEVs results in de-repressed TGFβR2 expression, enhanced TGF-β/pSmad2/3 signaling, and excessive collagen deposition. Administration of miR-194-3p mimics or agomiR-194-3p significantly reduced cardiac fibrosis in vivo and attenuated diabetic cardiomyopathy, establishing proof-of-concept for therapeutic EV-miRNA restoration [9]. This study elegantly demonstrates how diabetes disrupts the normal protective EV-miRNA communication between cardiac cell types.

#### 3.8.3. miR-21 and miR-126 in Cardiac Protection and Endothelial Function

In contrast to its pro-fibrotic role in the kidney, exosomal miR-21 derived from cardiac progenitor cells (CPCs) has demonstrated cardioprotective effects. CPC-derived exosomal miR-21 was shown to protect cardiomyocytes against apoptosis by targeting PDCD4, suggesting that the functional outcome of miR-21 signaling is highly dependent on the cellular context and the recipient cell type [78]. However, in the diabetic setting, the cardioprotective potential of CPC-derived exosomes may be impaired, as chronic hyperglycemia alters both the miRNA cargo and the functional capacity of exosomes released by cardiac stem/progenitor cells.

miR-126, a pivotal endothelial miRNA, is a critical regulator of angiogenesis and endothelial homeostasis. It is abundantly expressed in endothelial cell-derived EVs and targets VCAM-1 (vascular cell adhesion molecule 1) to suppress leukocyte adhesion and endothelial inflammation. In patients with T2DM, plasma miR-126 levels are significantly reduced, which correlates with endothelial dysfunction and increased cardiovascular risk [109]. Serum exosomal miR-126 levels were reported to be significantly decreased in patients with acute myocardial infarction, with an AUC of 0.822 for AMI diagnosis, while exosomal miR-21 was elevated (AUC = 0.856) [79]. The reciprocal regulation of these two exosomal miRNAs—one protective and one potentially pathogenic—provides a nuanced picture of EV-mediated cardiac biology in diabetes [110,111].

#### 3.8.4. Additional EV-miRNAs in Diabetic CVD Pathogenesis

Multiple additional EV-miRNAs have been implicated in the pathogenesis of cardiovascular complications in T2DM. Circulating exosomal miR-1 and miR-133a, which are highly expressed in cardiomyocytes and released upon myocardial injury, have been identified as biomarkers of myocardial steatosis in uncomplicated T2DM [112]. miR-181b-5p, carried in circulating EVs, influences vascular remodeling through modulation of the TGF-β/pSmad2/3 pathway and has been linked to diabetic vascular complications [113]. Exosomal miR-550a-5p and miR-665 have been identified as novel biomarkers for coronary microvascular dysfunction in diabetic patients, with mechanistic studies revealing their involvement in Hippo/eNOS signaling pathways that regulate endothelial function and microvascular integrity [106,114,115,116].

Circulating exosomal miR-16-2-3p was identified as a novel biomarker for coronary microvascular dysfunction in diabetes mellitus, with functional studies demonstrating its regulation of ACADM (acyl-CoA dehydrogenase medium chain) and fatty acid degradation pathways [117]. The involvement of fatty acid metabolism pathways in EV-miRNA-mediated cardiac pathology is particularly relevant given the lipotoxicity that characterizes the diabetic heart.

Diabetic cardiomyocyte-derived exosomes also carry miR-155, which has been shown to promote cardiac fibrosis by suppressing the Nrf2/HO-1 antioxidant pathway and upregulating collagen I and alpha-smooth muscle actin expression. In the atherosclerotic context, endothelial cell-derived EVs carrying altered miRNA cargo under diabetic conditions contribute to vascular smooth muscle cell phenotypic switching, foam cell formation, and plaque instability [3,118]. The comprehensive mapping of EV-miRNA communication networks in the diabetic cardiovascular system is an active and rapidly evolving area of research that promises to identify novel therapeutic targets and biomarker candidates.

### 3.9. EV-miRNAs as Diagnostic Biomarkers of CVDs in Type 2 Diabetes

The diagnostic application of EV-derived miRNAs in diabetic CVDs has progressed substantially in recent years, with several studies reporting individual miRNAs and multi-miRNA panels with clinically meaningful diagnostic accuracy. The following subsections summarize the most robust evidence to date [34,35,105,119].

#### 3.9.1. Ischemic Heart Disease Biomarkers

A systematic investigation of extracellular vesicle miRNAs as diagnostic and predictive biomarkers for ischemic heart disease (IHD) in patients with diabetes mellitus was conducted [29]. Through comprehensive profiling of serum EV-miRNAs, three candidates demonstrated exceptional diagnostic performance: miR-155-5p achieved an AUC of 0.901 with sensitivity of 84.3% and specificity of 86.5%; miR-15a-3p yielded an AUC of 0.874 with 79.6% sensitivity and 82.1% specificity; and miR-18a-5p demonstrated an AUC of 0.871 with 76.8% sensitivity and 80.3% specificity [29]. These AUC values exceed the 0.80 threshold generally considered clinically useful, suggesting that these EV-miRNAs could complement conventional cardiac biomarkers such as troponins and NT-proBNP in the diabetic population. The mechanistic relevance of these miRNAs is well-established: miR-155-5p regulates inflammatory signaling through SOCS1/NF-κB, miR-15a-3p modulates VEGF-A-mediated angiogenesis, and miR-18a-5p targets CTGF in fibrosis pathways [120].

#### 3.9.2. Carotid Atherosclerosis Detection

A rigorous discovery-validation study design was employed to identify serum exosomal miRNA biomarkers for carotid atherosclerosis (CAS) in patients with T2DM [20]. In the discovery phase, microarray analysis of serum exosomes from 12 T2DM patients (6 with CAS, 6 without) identified 23 differentially expressed miRNAs. Validation in 187 T2DM patients (training and validation cohorts) confirmed four miRNAs—hsa-miR-433-3p (AUC = 0.756), hsa-let-7b (AUC = 0.684), hsa-miR-30-5p (AUC = 0.721), and hsa-miR-122-5p (AUC = 0.699)—as significantly elevated in patients with CAS and positively correlated with carotid intima-media thickness (CIMT). The combined four-miRNA panel achieved a superior AUC of 0.833, outperforming individual miRNAs and demonstrating the added value of multi-miRNA panels for complex disease diagnosis [30].

Importantly, the diagnostic accuracy of this panel was maintained in an independent validation cohort (combined AUC = 0.816), and the panel was effective for detecting early-stage CAS (CIMT 1–1.5 mm, AUC = 0.79), suggesting potential utility for subclinical atherosclerosis screening in the diabetic population. Correlation analyses revealed associations between specific miRNAs and metabolic parameters: miR-433-3p, let-7b, and miR-30-5p correlated with HbA1c, while miR-122-5p correlated with LDL-C levels, suggesting these miRNAs may capture metabolic dimensions of cardiovascular risk not fully reflected by traditional biomarkers [30].

#### 3.9.3. Coronary Microvascular Dysfunction Biomarkers

Coronary microvascular dysfunction (CMD) is a particularly challenging diagnostic entity in diabetes, as it underlies anginal symptoms in the absence of obstructive epicardial coronary disease and is associated with adverse cardiovascular outcomes. Circulating exosomal miR-550a-5p and miR-665 were identified as novel biomarkers for CMD in diabetic patients, with significant diagnostic accuracy and mechanistic links to Hippo/eNOS signaling [114]. Separately, exosomal miR-16-2-3p was demonstrated to be dysregulated in CMD associated with diabetes, targeting ACADM in the fatty acid degradation pathway [117]. These findings are clinically significant because CMD in diabetes currently lacks reliable non-invasive biomarkers, and early detection could enable targeted interventions [121].

#### 3.9.4. Acute Myocardial Infarction and Heart Failure

Serum exosomal miR-21 and miR-126 have been evaluated as biomarkers for acute myocardial infarction (AMI), with reported AUCs of 0.856 and 0.822, respectively, and demonstrated correlation with cardiac troponin levels and PTEN expression [79]. In the heart failure domain, emerging evidence suggests that exosomal hsa-miR-339-5p may serve as a novel non-invasive biomarker, though validation in diabetic populations specifically is still needed [122]. Differential expression profiles of plasma exosomal miRNAs in diabetic cardiomyopathy were characterized, identifying distinct miRNA signatures that could distinguish diabetic cardiomyopathy from both healthy controls and patients with diabetes without cardiac involvement [104,123,124,125,126,127,128].

**Figure 2:**
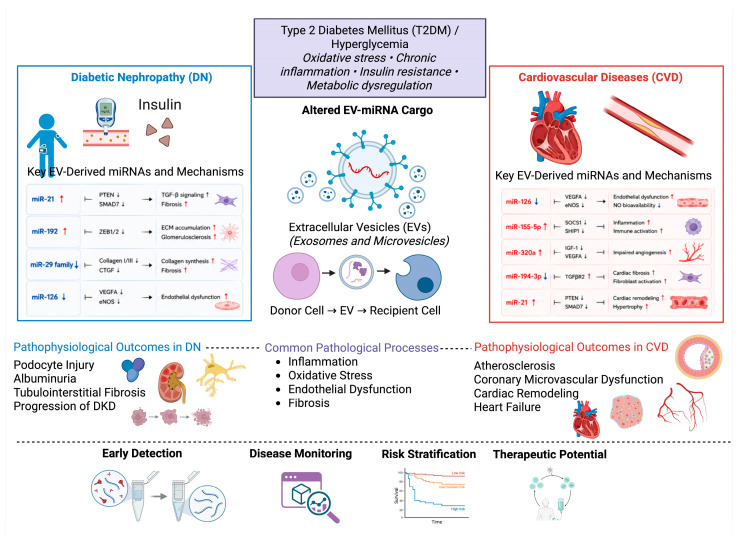
Mechanistic roles of EV-derived miRNAs in diabetic nephropathy and cardiovascular diseases associated with type 2 diabetes mellitus. Created with Created in BioRender.com. Abaildayev A. (2026), License No. GY29SLL9ES.

The emerging paradigm of EV-miRNA biomarkers for diabetic CVD underscores the advantages of multi-miRNA panels over single-miRNA approaches for achieving clinically useful diagnostic accuracy. The combination of miRNAs reflecting different pathogenic pathways (inflammation, fibrosis, angiogenesis, metabolic dysfunction) within a single panel may capture the multifaceted nature of diabetic cardiovascular disease more comprehensively than any individual biomarker. Furthermore, the integration of EV-miRNA panels with established clinical risk factors and imaging biomarkers holds promise for developing precision diagnostic algorithms tailored to the diabetic population. The diagnostic accuracy metrics for key EV-miRNA biomarkers of CVD in T2DM, including AUC, sensitivity, and specificity, are compiled in (Table 3) [13,34,35,129,130]. The major pathogenic pathways and biomarker applications of EV-derived miRNAs in DN and CVDs associated with T2DM are summarized in Figure 2.

A comprehensive synthesis of clinical studies on EV-miRNA biomarkers across all T2DM complications between 2020 and 2026 is compiled in Table 4.

### 3.10. Methodological Aspects of EV Isolation and miRNA Detection

The reliability and clinical utility of EV-miRNA biomarkers are fundamentally dependent on the technical methodologies employed for EV isolation, characterization, and miRNA quantification. Significant variability in reported EV-miRNA profiles across studies can often be attributed to differences in these methodological aspects, underscoring the critical need for standardization in the field [36,37,38,39,62].

#### 3.10.1. EV Isolation Methods: Comparative Analysis

Several EV isolation techniques are currently in use, each with distinct principles, advantages, and limitations. Ultracentrifugation (UC) at 100,000× *g* for 2–4 h has historically been considered the gold standard method, offering good purity for research applications but suffering from low throughput, requirement for expensive equipment, and co-isolation of protein aggregates and lipoproteins that may confound downstream miRNA analyses [36,38,123,132,133].

Size exclusion chromatography (SEC) has emerged as a preferred method for clinical biomarker studies, offering a favorable balance of EV purity, structural integrity preservation, and practical scalability. SEC separates EVs from smaller plasma components based on hydrodynamic radius using porous resin columns, and the resulting EV preparations show minimal contamination with soluble proteins while maintaining vesicle integrity for downstream functional and molecular analyses [36]. Density gradient ultracentrifugation (GRAD), using iodixanol or sucrose, provides the highest purity by separating EVs based on their buoyant density (1.13–1.19 g/mL), effectively excluding lipoproteins that float at different densities. A systematic comparison of SEC, GRAD, and combined SEC + GRAD methods demonstrated that GRAD yielded the highest reproducibility and miRNA diversity when combined with NEBNext library preparation for sequencing [36].

Polymer-based precipitation methods (e.g., ExoQuick, Total Exosome Isolation) offer simplicity and high yield but suffer from low specificity, co-precipitating non-EV proteins and lipoproteins. Immunoaffinity capture using antibody-coated magnetic beads targeting EV surface markers (CD63, CD9, CD81, EpCAM) provides the highest specificity for isolating specific EV subtypes but is expensive, low-throughput, and introduces bias toward the targeted EV population [38,134]. Tangential flow filtration (TFF) represents a scalable approach suitable for large-volume processing and is particularly relevant for therapeutic EV manufacturing. A detailed comparison of all major EV isolation methods, including their purity, yield, time requirements, costs, advantages, and limitations for miRNA biomarker studies, is provided in Table 5 [39,132].

#### 3.10.2. miRNA Detection and Quantification Platforms

Quantitative reverse-transcription polymerase chain reaction (qRT-PCR) remains the gold standard for miRNA quantification due to its high sensitivity (single-copy detection), established protocols, and relatively low cost. However, qRT-PCR requires prior knowledge of target miRNAs, limiting its utility in discovery settings. A critical challenge in EV-miRNA qRT-PCR is normalization, as traditional reference genes (e.g., U6 snRNA, RNU48) may be variably present in EV preparations. The use of exogenous spike-in controls, particularly cel-miR-39 from Caenorhabditis elegans, has become a widely adopted normalization strategy [5,100,135,136].

Droplet digital PCR (ddPCR) offers absolute quantification of target miRNAs without the need for standard curves or reference genes, providing superior precision for rare targets and eliminating normalization challenges. Its partitioning-based approach also confers greater tolerance to PCR inhibitors present in clinical samples [137]. Next-generation sequencing (NGS/small RNA-seq) enables unbiased, comprehensive profiling of the entire small RNA transcriptome within EVs, including novel miRNAs and isomiRs. While NGS provides the most thorough discovery platform, it requires substantial bioinformatics expertise, is susceptible to library preparation biases, and has higher per-sample costs [36,138].

The NanoString nCounter system offers a middle ground, enabling direct digital detection of up to 800 miRNA targets simultaneously from low RNA input without amplification, thereby avoiding PCR bias [139]. Emerging technologies include localized surface plasmon resonance (LSPR) biosensors for rapid, label-free miRNA detection at the point of care, and CRISPR-based nucleic acid detection systems (SHERLOCK, DETECTR) that combine isothermal amplification with CRISPR collateral cleavage for highly sensitive and specific miRNA detection without sophisticated laboratory infrastructure. A systematic comparison of all major miRNA detection platforms, encompassing sensitivity, throughput, cost, and technical requirements, is presented in Table 6.

#### 3.10.3. Preanalytical Considerations and Quality Control

Preanalytical variables represent a major source of variability in EV-miRNA studies and must be rigorously controlled for clinical implementation. Sample collection parameters, including anticoagulant type (EDTA versus citrate), needle gauge, tourniquet time, hemolysis, and time-to-processing, significantly influence both EV yield and miRNA profiles. For urinary EV studies, factors such as collection timing (first morning void versus random), protease inhibitor addition, centrifugation speed, and storage temperature must be standardized [38,140,141]. The ISEV MISEV2023 guidelines provide comprehensive recommendations for sample handling, EV characterization (including requirements for demonstrating EV-associated versus non-EV-associated markers), and reporting standards that should be adopted by all biomarker studies to facilitate cross-study comparisons and eventual clinical validation [37,142].

### 3.11. Clinical Translation and Challenges

Despite the compelling preclinical and clinical evidence supporting EV-miRNAs as biomarkers for DN and CVDs, the translation of these findings into routine clinical practice faces substantial challenges that must be systematically addressed [11,12,143,144].

Patient heterogeneity, including differences in ethnicity, medication use, glycemic control, and renal function stage, together with methodological variability in sample handling, EV isolation, and normalization strategies, may substantially influence EV-miRNA profiles and contribute to differences in reported AUC, sensitivity, and specificity across studies. Therefore, greater standardization is required to improve reproducibility and clinical translation.

#### 3.11.1. Standardization of Preanalytical and Analytical Protocols

Perhaps the most significant barrier to clinical translation is the lack of standardized protocols for EV isolation, miRNA extraction, and quantification. As discussed in Section 3.9, different isolation methods yield EV preparations with distinct compositions, purity levels, and miRNA profiles, making cross-study comparisons unreliable. The variability extends to preanalytical factors including sample collection, processing, and storage conditions. Until consensus protocols are established and widely adopted, the field will continue to be plagued by inconsistent results and limited reproducibility [36,37,140]. International efforts, including the ISEV MISEV guidelines and the Extracellular RNA Communication Consortium (ERCC), are working toward standardization, but widespread adoption remains incomplete. 

#### 3.11.2. Normalization Challenges

The absence of universally accepted endogenous reference genes for EV-miRNA normalization is a critical technical challenge. Unlike cellular miRNA studies where U6 snRNA or other small nuclear RNAs can serve as stable references, these molecules may be inconsistently present in EV preparations depending on the isolation method. Various normalization strategies have been employed across studies, including exogenous spike-in controls (cel-miR-39), geometric mean of multiple endogenous miRNAs, global mean normalization for high-throughput platforms, and normalization to EV particle count. Each approach has merits and limitations, and the lack of consensus undermines the comparability of diagnostic thresholds across studies [135,138,145].

#### 3.11.3. Cohort Heterogeneity and Study Design Limitations

The majority of published studies are single-center, case–control studies with relatively small sample sizes, which are prone to selection bias, overfitting, and limited generalizability. The heterogeneity in patient populations—regarding diabetes duration, glycemic control, concomitant medications (particularly SGLT2 inhibitors and GLP-1 receptor agonists, which may independently affect EV biology), comorbidities, and ethnic diversity—complicates the identification of robust, universally applicable biomarkers. Furthermore, many studies use albuminuria-based rather than biopsy-based classification of DN, introducing diagnostic misclassification bias. The field urgently requires large-scale, prospective, multicenter studies with standardized protocols, well-characterized cohorts, and long-term follow-up to establish the true clinical utility of EV-miRNA biomarkers [26,146].

#### 3.11.4. Tissue Specificity and EV Heterogeneity

Circulating EVs represent a heterogeneous mixture of vesicles derived from multiple cell types and tissues, making it challenging to attribute specific miRNA changes to a particular organ or disease process. While urinary EVs are enriched for kidney-derived vesicles, they also contain EVs from the urinary tract epithelium and potentially from filtered plasma. Serum/plasma EVs are contributed by platelets, erythrocytes, endothelial cells, immune cells, and multiple organs, creating a complex mixture in which disease-specific signals may be diluted [52,147,148]. Strategies to overcome this limitation include the use of organ-specific surface markers for immunoaffinity capture (e.g., podocalyxin for podocyte-derived EVs, troponin T for cardiomyocyte-derived EVs), EV surface protein profiling by single-vesicle analysis technologies, and computational deconvolution approaches that infer tissue-of-origin from multi-omics EV profiles [63,136].

#### 3.11.5. Integration with Existing Clinical Workflows

For EV-miRNA biomarkers to achieve clinical adoption, they must demonstrate added value over existing diagnostic tools and be implementable within current laboratory infrastructure. This requires developing standardized, user-friendly assay formats (ideally point-of-care compatible), establishing clinically validated cut-off values, demonstrating cost-effectiveness in health economic analyses, and obtaining regulatory approval through clinical trials meeting FDA or EMA requirements for in vitro diagnostic devices. The development of multiplexed assays capable of simultaneously measuring multiple EV-miRNA biomarkers in a single test, combined with machine learning algorithms for data integration, represents a promising path toward clinically actionable diagnostic tools [68,146,149].

### 3.12. Future Perspectives

The field of EV-miRNA biomarkers for diabetic complications stands at a critical juncture, with substantial foundational evidence accumulated but significant translational work remaining. Several key directions will shape the future trajectory of this field [11,19,33,150]. An additional challenge is that several EV-associated miRNAs, including miR-21, miR-192, and members of the miR-29 family, are dysregulated across multiple diabetic complications and therefore may lack sufficient disease specificity when used individually. Future diagnostic approaches will likely require multi-miRNA panels, tissue-specific EV populations, and integration with clinical parameters to accurately distinguish between renal and cardiovascular complications.

Another important consideration is the temporal stability of EV-miRNA biomarkers. Although EV-encapsulated miRNAs are generally stable in biological samples due to protection by the vesicular membrane, their expression levels may fluctuate in response to glycemic control, disease progression, inflammation, and therapeutic interventions. Emerging evidence suggests that improvements in glycemic control and treatment may alter EV-miRNA expression profiles over time, indicating that these biomarkers may reflect dynamic disease activity rather than remain completely stable throughout the course of diabetes. Therefore, longitudinal studies are needed to evaluate intra-individual variability and determine their utility for disease monitoring and treatment response assessment.

Beyond issues of disease specificity and temporal variability, limited reproducibility remains a major obstacle to the clinical implementation of EV-miRNA biomarkers. Although numerous EV-associated miRNAs, including miR-21, miR-29 family members, miR-126, miR-192, and miR-155, have demonstrated promising diagnostic potential in individual studies, relatively few biomarkers have been consistently validated across independent cohorts and different clinical settings. Consequently, no universally accepted EV-miRNA biomarker panel has yet emerged for DN or cardiovascular complications in T2DM. This lack of reproducibility likely reflects both biological heterogeneity among patient populations and methodological differences in EV isolation, miRNA detection, normalization strategies, and study design. Therefore, large multicenter studies with standardized protocols and external validation cohorts are required to establish robust and clinically applicable EV-miRNA biomarkers.

#### 3.12.1. Multi-Omics Integration and Systems Biology Approaches

Future biomarker studies should move beyond single-miRNA or even multi-miRNA panels to embrace multi-omics approaches that integrate EV-miRNA profiles with EV proteomics, lipidomics, and metabolomics data. The combination of different molecular layers within a single EV analysis could capture complementary information about disease biology, potentially improving diagnostic accuracy and providing mechanistic insights. Systems biology frameworks that model the interactions between EV-miRNAs and their target networks in the context of DN and CVD could identify novel biomarker combinations and therapeutic targets that are not apparent from single-molecule studies [10,34,65,142].

#### 3.12.2. Single-Vesicle Analysis Technologies

The heterogeneity of EV populations within a sample presents both a challenge and an opportunity. Emerging single-vesicle analysis technologies, including nano-flow cytometry, single-particle interferometric reflectance imaging sensing (SP-IRIS), and droplet-based microfluidic platforms, enable the characterization of individual EVs in terms of their surface markers, size, and molecular cargo. These technologies could resolve tissue-specific EV subpopulations within complex biological fluids, allowing for the identification of disease-specific EV signatures with unprecedented precision [134,147]. The integration of single-vesicle protein and nucleic acid profiling could yield composite biomarkers that incorporate information about both the cellular source and the molecular cargo of individual EVs [151,152].

#### 3.12.3. Artificial Intelligence and Machine Learning

The high-dimensional nature of EV-miRNA datasets, particularly those generated by NGS platforms, makes them ideally suited for machine learning approaches. Deep learning algorithms can identify complex patterns in multi-miRNA profiles that may not be apparent through traditional statistical methods, potentially improving biomarker panel selection and diagnostic classification. Several groups have begun applying random forest, support vector machine, and neural network classifiers to EV-miRNA data for disease classification, with promising results in terms of prediction accuracy. Future work should focus on developing interpretable AI models that not only provide accurate diagnoses but also reveal the biological pathways underlying their predictions, facilitating mechanistic understanding and clinical trust [68,153,154].

#### 3.12.4. Theranostic Applications

The same EV-miRNAs that serve as diagnostic biomarkers may also represent therapeutic targets or delivery vehicles. The concept of theranostics—combining diagnostics and therapeutics—is particularly attractive in the EV-miRNA field. For example, the identification of reduced miR-194-3p delivery from cardiomyocytes to fibroblasts in diabetes simultaneously provides a biomarker of diabetic cardiac fibrosis and a therapeutic target (agomiR-194-3p delivery) [9]. Similarly, the restoration of EV-miR-29 family members in the diabetic kidney could both ameliorate fibrosis and serve as a pharmacodynamic biomarker of treatment response. Engineered EVs loaded with therapeutic miRNAs represent a promising drug delivery platform due to their inherent biocompatibility, ability to cross biological barriers, and potential for cell-type-specific targeting through surface engineering [68,153,154].

#### 3.12.5. Prospective Multicenter Clinical Trials

The most pressing need in the field is the execution of large-scale, prospective, multicenter clinical trials designed to validate EV-miRNA biomarker panels against clinical outcomes. These trials should employ standardized protocols for sample collection, EV isolation, and miRNA quantification; include diverse patient populations; incorporate longitudinal follow-up to assess prognostic utility; and evaluate the incremental value of EV-miRNA biomarkers over existing clinical algorithms. Collaboration between academic researchers, clinical centers, diagnostic companies, and regulatory agencies will be essential to design and execute trials that can generate the level of evidence required for clinical adoption and regulatory approval [9,155,156].

## 4. Conclusions

Extracellular vesicle-derived microRNAs (EV-miRNAs) have emerged as highly promising non-invasive biomarkers for the early detection and monitoring of DN and CVDs in patients with T2DM. Accumulating evidence demonstrates that EV-miRNAs reflect the molecular and pathophysiological alterations occurring in diabetic tissues, including renal fibrosis, endothelial dysfunction, inflammation, oxidative stress, and cardiac remodeling. Several EV-associated miRNAs, including miR-21, miR-192, miR-29 family members, miR-155-5p, miR-126, and miR-194-3p, have shown significant diagnostic and mechanistic relevance across diabetic renal and cardiovascular complications. Compared with conventional biomarkers such as albuminuria, eGFR, and circulating inflammatory markers, EV-miRNAs offer several advantages, including high stability in biological fluids, protection from RNase-mediated degradation, tissue-specific expression patterns, and the ability to capture early molecular changes before irreversible organ damage becomes clinically apparent. Importantly, urinary and circulating EV-miRNA panels demonstrated encouraging diagnostic accuracy in multiple studies, highlighting their potential utility within precision medicine and liquid biopsy approaches. Despite these advances, several important challenges remain before EV-miRNA biomarkers can be translated into routine clinical practice. These include the lack of standardized protocols for EV isolation and characterization, variability in miRNA normalization strategies, heterogeneity among patient cohorts, and limited large-scale prospective validation studies. Furthermore, differences in EV isolation techniques and analytical platforms continue to affect reproducibility across studies. Future investigations should prioritize multicenter longitudinal studies, standardized methodological frameworks in accordance with MISEV guidelines, and integration of EV-miRNA signatures with clinical, imaging, and multi-omics datasets. The application of artificial intelligence and machine learning approaches may further enhance the diagnostic and prognostic performance of EV-miRNA panels. Collectively, EV-derived miRNAs represent a rapidly evolving and clinically relevant biomarker platform with substantial potential to improve the early diagnosis, risk stratification, and personalized management of DN and CVDs in T2DM.

## Figures and Tables

**Table 1:** Classification, Biogenesis, and Characteristics of Extracellular Vesicle Subtypes.

EV Type	Size (nm)	Biogenesis Pathway	Surface Markers	Molecular Cargo	Main Function
Exosomes	30–150	Endosomal pathway: MVBs formed via ESCRT-dependent or ceramide/tetraspanin-dependent ILV budding; MVB fusion with plasma membrane releases ILVs as exosomes	CD63, CD9, CD81, TSG101, Alix, Flotillin-1, Syntenin-1	miRNAs, lncRNAs, mRNAs, proteins, lipids, metabolites	Intercellular communication, immune modulation, antigen presentation, waste removal
Microvesicles (Ectosomes)	100–1000	Direct outward budding and fission from plasma membrane; involves ARF6, RHOA, cytoskeletal reorganization, calcium-dependent enzymes	Integrins, Selectins, CD40 ligand, ARF6, Annexin V, Tissue Factor	miRNAs, mRNAs, cytoplasmic proteins, surface receptors, organelles	Coagulation, inflammation, cell signaling, extracellular matrix remodeling
Apoptotic Bodies	1000–5000	Released during programmed cell death; membrane blebbing and cellular disassembly during late apoptosis	Phosphatidylserine (outer leaflet), Annexin V, C3b, Thrombospondin	Nuclear fragments, DNA, histones, organelles, cytoplasmic content	Clearance of dying cells, immune regulation, horizontal gene transfer

**Table 2:** Key EV-Derived miRNAs as Biomarkers in Diabetic Nephropathy (DKD).

miRNA	Regulation	Sample Source	Study Type	Patient Cohort (*n*)	AUC	Sn (%)	Sp (%)	Target Genes/Pathways	Reference (Year)
miR-4534	↑	Urinary exosomes	Case–control	DKD = 14, DM = 14	0.786	NR	NR	BNIP3/FoxO signaling pathway	Zhao et al. (2020) [5]
miR-4449	↑	Serum exosomes	Case–control	DKD = 30, HC = 30	NR	NR	NR	PHRF1/pyroptosis/NLRP3 inflammasome	Gao et al. (2021) [85]
miR-151a-3p	↑ in DN	Urinary ECVs	Case–control	T2D + DN = 10, T2D-DN = 11, HC = 10	NR	NR	NR	EGFR, MAP3K signaling	Ali et al. (2024) [102]
miR-182-5p	↑ in DN	Urinary ECVs	Case–control	T2D + DN = 10, T2D-DN = 11, HC = 10	NR	NR	NR	FOXO1, RECK, MTSS1	Ali et al. (2024) [102]
miR-136-5p	↑	Urinary exosomes	Case–control	DN = 42, T2DM = 38	0.722	72.2	78.4	PTEN/PI3K/Akt signaling	Zheng et al. (2025) [100]
miR-142-3p	↑	Urinary exosomes	Two-phase validation	DN = 57, T2DM = 42	Significant	NR	NR	Fatty acid metabolism, Hippo, Wnt	Li et al. (2025) [89]
miR-183-5p	↑	Urinary exosomes	Case–control	T2DM = 27, HC = 15	NR	NR	NR	mTOR, FoxO signaling	Fang et al. (2024) [103]
miR-125a-5p	↓	Urinary exosomes	Case–control	T2DM = 27, HC = 15	NR	NR	NR	p53, cell cycle regulation	Fang et al. (2024) [103]
miR-663a	↑	Urinary exosomes	Case–control	DKD = 33, T2DM = 27	0.71	NR	NR	Proximal tubular injury markers	Sinha et al. (2023) [101]
miR-483-5p	↓	Urinary exosomes	Sequencing + validation	DKD = biopsy confirmed	NR	NR	NR	MAPK1/TIMP2 → fibrosis	Li et al. (2025) [89]
miR-192	↑	Urinary exosomes	Systematic review	Multiple cohorts	0.79 *	0.76 *	0.74 *	TGF-β/Smad3/ZEB1/ZEB2/Col1a2	Wang et al. (2025) [104]
Pooled miRNAs	Various	Serum/Urine	Meta-analysis (24 studies)	*n* = 3052	0.79	76	74	Multiple pathways	Wang et al. (2025) [104]
miR-145-5p	↑	Urinary exosomes	Case–control	DKD cohort	NR	NR	NR	Srgap2/RhoA/ROCK → podocyte apoptosis	Han et al. (2023) [90]
miR-320c	↑	Urinary exosomes	Case–control	DKD with microalbuminuria	NR	NR	NR	TSP-1/TGF-β signaling	Delić et al. (2016) [97]

Note: ↑ indicates upregulation (increased expression), whereas ↓ indicates downregulation (decreased expression). * Values reported from a systematic review/meta-analysis.

**Table 3:** Key EV-Derived miRNAs as Biomarkers in CVDs in T2DM.

miRNA	Regulation	CVD Condition	Sample Source	AUC	Sn (%)	Sp (%)	Molecular Target	Reference (Year)
miR-155-5p	↑	Ischemic Heart Disease in DM	Serum EVs	0.901	84.3	86.5	SOCS1/NF-κB inflammatory signaling	Li et al. (2022) [28]
miR-15a-3p	↑	Ischemic Heart Disease in DM	Serum EVs	0.874	79.6	82.1	VEGF-A/angiogenesis pathway	Li et al. (2022) [28]
miR-18a-5p	↑	Ischemic Heart Disease in DM	Serum EVs	0.871	76.8	80.3	CTGF/fibrosis signaling	Li et al. (2022) [28]
miR-433-3p	↑	Carotid Atherosclerosis in T2DM	Serum exosomes	0.756	NR	NR	Vascular calcification pathways	Lu et al. (2026) [30]
let-7b	↑	Carotid Atherosclerosis in T2DM	Serum exosomes	0.684	NR	NR	Coronary stenosis severity	Lu et al. (2026) [30]
miR-30-5p	↑	Carotid Atherosclerosis in T2DM	Serum exosomes	0.721	NR	NR	Cardiac remodeling	Lu et al. (2026) [30]
miR-122-5p	↑	Carotid Atherosclerosis in T2DM	Serum exosomes	0.699	NR	NR	LDL metabolism, plaque instability	Lu et al. (2026) [30]
Panel (4 miRNAs)	↑	CAS in T2DM	Serum exosomes	0.833	NR	NR	Combined: CIMT correlation	Lu et al. (2026) [30]
miR-550a-5p	Altered	Coronary Microvascular Dysfunction	Circulating exosomes	Significant	NR	NR	Hippo/eNOS signaling	Ding et al. (2026) [114]
miR-665	Altered	Coronary Microvascular Dysfunction	Circulating exosomes	Significant	NR	NR	Hippo/eNOS signaling	Ding et al. (2026) [114]
miR-16-2-3p	↑	Coronary Microvascular Dysfunction in DM	Circulating exosomes	Significant	NR	NR	ACADM/fatty acid degradation	Liu et al. (2024) [117]
miR-21	↑	Acute Myocardial Infarction	Serum exosomes	0.856	80.0	78.5	PDCD4/apoptosis, PTEN	Ling et al. (2020) [79]
miR-126	↓	AMI/Endothelial dysfunction	Serum exosomes	0.822	76.3	75.0	VCAM-1, VEGF, angiogenesis	Ling et al. (2020) [79]
miR-194-3p	↓	Diabetic Cardiomyopathy/Cardiac Fibrosis	Cardiomyocyte sEVs	N/A (mechanistic)	N/A	N/A	TGFβR2 → fibroblast-myofibroblast conversion	Li et al. (2024) [31]
miR-320	↑	Diabetic Cardiomyopathy	Cardiomyocyte exosomes	N/A (mechanistic)	N/A	N/A	IGF-1, HSP20, Ets2 → anti-angiogenic	Wang et al. (2014) [107]

Note: ↑ indicates upregulation (increased expression), whereas ↓ indicates downregulation (decreased expression).

**Table 4:** Summary of Clinical Studies on EV-miRNA as Biomarkers in T2DM Complications (2020–2026).

Author (Year)	Study Design	Sample Type	miRNA(s)	T2DM Complication	*n* (Cases/Controls)	Key Finding	AUC	Ref
Zhao et al. (2020)	Case–control	Urinary exosomes	miR-4534	DKD	14/14	miR-4534 upregulated; correlated with UALB; targets BNIP3/FoxO pathway	0.786	[5]
Gao et al. (2021)	Case–control	Serum exosomes	miR-4449	DKD	30/30	Promotes pyroptosis via PHRF1; induces oxidative stress in tubular cells	NR	[85]
Ali et al. (2024)	Case–control	Urinary ECVs	miR-151a-3p, miR-182-5p	DN	10 DN/11 T2D/10 HC	Distinguish T2D + DN from T2D-DN; 13 dysregulated miRNAs identified via NGS	NR	[102]
Fang et al. (2024)	Case–control	Urinary exosomes	miR-183-5p, miR-125a-5p	T2DM kidney	27/15	miR-183-5p upregulated, miR-125a-5p downregulated in T2DM	NR	[103]
Wang et al. (2025)	Case–control	Urinary exosomes	miR-136-5p	DKD	42 DN/38 T2DM	First report of miR-136-5p upregulation in DKD urinary exosomes	0.722	[111]
Zhou et al. (2025)	Sequencing + validation	Urinary exosomes	miR-483-5p (↓), others	DKD (biopsy)	Biopsy-confirmed	miR-483-5p downregulated; promotes podocyte apoptosis and fibrosis	NR	[89]
Sinha et al. (2023)	Case–control	Urinary exosomes	miR-663a	DKD	33/27	Proximal tubular injury marker; correlated with eGFR decline	0.71	[101]
Wang et al. (2025)	Meta-analysis	Serum/Urine	Pooled (24 studies)	DKD	*n* = 3052	Pooled diagnostic performance: Sn = 0.76, Sp = 0.74	0.79	[104]
Ling et al. (2020)	Case–control	Serum exosomes	miR-21, miR-126	AMI	AMI patients/HC	miR-21 ↑ and miR-126 ↓ in AMI; correlated with cardiac troponin	0.856/0.822	[79]
Li et al. (2022)	Case–control	Serum EVs	miR-155-5p, miR-15a-3p, miR-18a-5p	IHD in DM	IHD-DM/DM/HC	Three miRNAs with excellent diagnostic performance for IHD-DM	0.871–0.901	[28]
Zhang et al. (2023)	Case–control	Plasma exosomes	Multiple miRNAs	DCM	DCM/T2DM/HC	Distinct exosomal miRNA profiles in diabetic cardiomyopathy	NR	[131]
Li et al. (2024)	Preclinical + mechanistic	Cardiomyocyte sEVs	miR-194-3p	DCM/Cardiac fibrosis	db/db mice, HFD mice	miR-194-3p reduced in diabetic sEVs; targets TGFβR2; agomiR rescues fibrosis	N/A	[31]
Liu et al. (2024)	Case–control	Circulating exosomes	miR-16-2-3p	CMD in DM	CMD-DM/DM/HC	Novel biomarker for coronary microvascular dysfunction; targets ACADM	Significant	[117]
Ding et al. (2026)	Case–control	Circulating exosomes	miR-550a-5p, miR-665	CMD in DM	CMD patients	Identified Hippo/eNOS signaling as mechanistic link	Significant	[114]
Lu (2026)	Discovery-validation	Serum exosomes	miR-433-3p, let-7b, miR-30-5p, miR-122-5p	CAS in T2DM	58/129 (train + valid)	Four-miRNA panel for CAS diagnosis; correlated with CIMT severity	0.833	[30]
Han et al. (2023)	Case–control + in vitro	Urinary exosomes	miR-145-5p	DKD	DKD cohort	Induces podocyte apoptosis via Srgap2/RhoA/ROCK pathway	NR	[90]

Note: ↑ indicates upregulation (increased expression), whereas ↓ indicates downregulation (decreased expression). 73DKD, diabetic kidney disease; DN, diabetic nephropathy; AMI, acute myocardial infarction; IHD, ischemic heart disease; DCM, diabetic cardiomyopathy; CMD, coronary microvascular dysfunction; CAS, carotid atherosclerosis; HC, healthy controls; NR, not reported; NGS, next-generation sequencing.

**Table 5:** Comparison of EV Isolation Methods for miRNA Biomarker Studies.

Method	Principle	Purity	Yield	Time	Cost	Advantages	Limitations	Recommended Use
Ultracentrifugation (UC)	Sequential centrifugation at 100,000× *g* for 2–4 h sediments EVs by density	Moderate	Low–Moderate	4–6 h	High (equipment)	Gold standard; well-established protocols; suitable for large volumes	Time-consuming; requires ultracentrifuge; co-isolation of protein aggregates and lipoproteins; low throughput	Research; large volume samples
Size Exclusion Chromatography (SEC)	Separation by hydrodynamic radius through porous resin columns (e.g., Sepharose CL-2B)	High	Moderate–High	30–60 min	Moderate	Good purity; preserves EV integrity; scalable; gentle; reproducible	Co-elution with similarly sized lipoproteins; requires column equilibration; limited concentration	Clinical biomarker studies; plasma/serum
Density Gradient (GRAD)	Iodixanol or sucrose gradient separates EVs by buoyant density (1.13–1.19 g/mL)	Very High	Low	16–18 h	High	Highest purity; best lipoprotein separation; excellent for miRNA profiling	Very time-consuming; low yield; complex protocol; not scalable	Discovery studies; high-purity miRNA sequencing
Polymer Precipitation	PEG-based polymers (ExoQuick, TEI) reduce solubility, precipitating EVs	Low	Very High	30 min–overnight	Low	Simple; fast; no special equipment; high yield; suitable for large cohorts	Co-precipitation of non-EV proteins and lipoproteins; low specificity; high background	High-throughput screening; preliminary studies
Immunoaffinity Capture	Antibody-coated magnetic beads targeting EV surface markers (CD63, CD9, CD81, EpCAM)	Very High	Low	2–4 h	Very High	Highest specificity; subtype isolation; excellent for targeted analyses	Expensive; limited scalability; bias toward specific EV populations; antibody availability	Subtype-specific studies; mechanistic research
SEC + GRAD Combined	Sequential SEC followed by density gradient fractionation	Highest	Low	18–24 h	Very High	Optimal purity for clinical miRNA biomarker studies; best reproducibility	Very time-consuming; complex; low throughput; requires expertise	Clinical miRNA biomarker validation
Tangential Flow Filtration (TFF)	Membrane-based filtration using defined pore sizes under tangential flow	Moderate–High	High	1–3 h	High (equipment)	Scalable; gentle; suitable for large volumes; GMP-compatible	Requires specialized equipment; membrane fouling; not widely standardized	Large-scale production; therapeutic EV preparation

**Table 6:** Comparison of EV-miRNA Detection and Quantification Methods.

Method	Sensitivity	Specificity	Throughput	Cost	Technical Requirements	Advantages	Limitations
qRT-PCR	Very High (single copy)	High (primer-specific)	Low (single/multiplex)	Low–Moderate	Standard lab; thermal cycler; requires normalization (e.g., cel-miR-39 spike-in)	Gold standard; high sensitivity and reproducibility; well-established; quantitative	Requires prior knowledge of targets; limited multiplexing; normalization challenges; not suitable for discovery
Droplet Digital PCR (ddPCR)	Very High (absolute)	Very High	Low–Moderate	High	Specialized ddPCR system; droplet generator	Absolute quantification without calibration curves; no normalization needed; robust to inhibitors	Expensive equipment; limited multiplexing (2–4 targets); requires specialized reagents
Small RNA-seq (NGS)	High	High (genome-wide)	Very High	Very High	Sequencing platform (Illumina); bioinformatics expertise; library preparation	Unbiased discovery; novel miRNA identification; comprehensive profiling; isomiR detection	High cost; complex data analysis; sequencing bias from library preparation; requires high RNA input
NanoString nCounter	Moderate–High	High	High (800 targets)	High	nCounter instrument; probe design	Direct digital detection; no amplification bias; low RNA input; multiplex (up to 800 miRNAs)	Fixed probe panel; cannot discover novel miRNAs; moderate sensitivity; proprietary platform
Microarray	Moderate	Moderate	Very High	Moderate	Array scanner; hybridization equipment	High throughput; genome-wide coverage; well-established; relatively low cost per target	Cross-hybridization; limited dynamic range; requires validation by qRT-PCR; not absolute quantification
LSPR Biosensors	High	High	High (point-of-care)	Moderate (development)	Nanoplasmonic chip; optical reader	Rapid; label-free; potential for point-of-care; multiplexing possible; minimal sample preparation	Emerging technology; limited validation; requires specialized fabrication; not yet standardized
In Situ Hybridization (ISH)	Moderate	Very High	Very Low	Moderate	Microscopy; fluorescent/chromogenic probes	Spatial localization in tissues; confirms cellular source of miRNAs; can combine with immunostaining	Low throughput; semi-quantitative; technically demanding; not suitable for liquid biopsy screening

## Data Availability

No new data were created or analyzed in this study. Data sharing is not applicable to this article.

## Abbreviations

ACADM	Acyl-CoA Dehydrogenase Medium Chain
AGEs	Advanced Glycation End Products
AMI	Acute Myocardial Infarction
ApoBDs	Apoptotic Bodies
AUC	Area Under the Receiver Operating Characteristic Curve
CAS	Carotid Atherosclerosis
CVD	Cardiovascular Disease
DCM	Diabetic Cardiomyopathy
DKD	Diabetic Kidney Disease
DN	Diabetic Nephropathy
ECM	Extracellular Matrix
EVs	Extracellular Vesicles
EMT	Epithelial-to-Mesenchymal Transition
ISEV	International Society for Extracellular Vesicles
miRNAs	MicroRNAs
NGS	Next-Generation Sequencing
T2DM	Type 2 Diabetes Mellitus
